# Cascade screening for beta-thalassaemia in Pakistan: relatives’ experiences of a decision support intervention in routine practice

**DOI:** 10.1038/s41431-021-00974-y

**Published:** 2021-10-04

**Authors:** Shenaz Ahmed, Hussain Jafri, Muhammed Faran, Wajeeha Naseer Ahmed, Yasmin Rashid, Yasmin Ehsan, Mushtaq Ahmed

**Affiliations:** 1 Leeds Institute of Health Sciences, University of Leeds, Leeds, UK; 2 Thalassaemia Society Pakistan, Lahore, Pakistan; 3 Fatima Jinnah Medical University, Lahore, Pakistan; 4 Health Department Punjab, Lahore, Pakistan; 5 Yorkshire Regional Genetics Service, Leeds, UK

**Keywords:** Patient education, Genetic counselling

## Abstract

Low uptake of cascade screening for βeta-thalassaemia major (β-TM) in the ‘Punjab Thalassaemia Prevention Project’ (PTPP) in Pakistan led to the development of a ‘decision support intervention for relatives’ (DeSIRe). This paper presents the experiences of relatives of children with β-TM of the DeSIRe following its use by PTPP field officers (FOs) in routine clinical practice. Fifty-four semi-structured qualitative interviews were conducted (April to June 2021) with relatives in seven cities in the Punjab province (Lahore, Sheikhupura, Nankana Sahab, Kasur, Gujranwala, Multan and Faisalabad). Thematic analysis shows that participants were satisfied with the content of the DeSIRe and its delivery by the FOs in a family meeting. They understood that the main purpose of the DeSIRe was to improve their knowledge of β-TM and its inheritance, and to enable them to make decisions about thalassaemia carrier testing, particularly before marriage. Participants also raised concerns about the stigma of testing positive; however, they believed the DeSIRe was an appropriate intervention, which supported relatives to make informed decisions. Our findings show that the DeSIRe is appropriate for use by healthcare professionals in routine practice in a low-middle income country, and has the potential to facilitate shared decision making about cascade screening for thalassaemia. Further research is needed to prove the efficacy of the DeSIRe.

## Introduction

The World Health Organization has designated the prevention of βeta-thalassaemia major (β-TM) in developing countries a priority. Pakistan has a population of approximately 225 million people and a β-TM carrier frequency of more than 5%, so over 9 million carriers [1]. β-TM is the most common genetic disorder in Pakistan, with an estimated 40,000 children registered as transfusion-dependent and 5000–9000 children born annually with the condition [2].

The Punjab Thalassaemia Prevention Project (PTPP) is a government-funded provincial intervention to reduce the incidence of β-TM. One of its priorities is to facilitate cascade screening, to identify and offer thalassaemia carrier testing to biological relatives to enable them to make informed marital and reproductive decisions. The PTPP has nine regional centres that cover all 36 districts of Punjab province. For cascade screening, the PTPP’s 48 field officers (FOs) approach parents of children with β-TM in thalassaemia clinics, who in turn arrange a family meeting at their home to enable FOs to provide genetic counselling. However, uptake rates of cascade screening are suboptimal, suggesting the need for information resources to support relatives’ decision making.

Decision support interventions (also known as decision aids) can support relatives to make decisions, by making their decisions explicit, providing information about the condition, testing options and associated benefits/harms and helping clarify personal values [3]. Decision support interventions can also facilitate shared decision making (SDM). FOs and relatives can make decisions together using evidence, where relatives are supported to consider available screening, and the likely benefits and harms of this [4]. SDM with relatives is particularly important because the decision about cascade screening is partly dependent on their values and preferences for particular outcomes, where there is little scientific evidence for the advantages of the options, and respect for individuals’ autonomy in this context of genetic counselling is paramount [5].

Studies show that people using decision aids feel more knowledgeable, better informed and clearer about their values [3, 6], and, therefore, are likely to make informed values-based decisions [7]. Furthermore, people usually prefer to be included in decision making, as opposed to paternalistic or just autonomous approaches [8]. However, most of the research on using decision support interventions or SDM has been conducted in western countries, and may not be transferable to low-middle income countries (LMICs). For example, healthcare professionals in Pakistan are unlikely to engage in SDM due to sociocultural factors, including low level of education, patriarchal family systems and patient expectations for doctors to direct them rather than facilitate medical decisions [9, 10]. Our research in Pakistan also shows people’s preferences for directive advice for medical decision making [11]. However, we are not aware of any research in Pakistan to explore the development or use of a decision support intervention. Any such intervention developed specifically for use in Pakistan may be acceptable. Therefore, we developed a decision support intervention for relatives (DeSIRe) of children with β-TM.

The paper-based DeSIRe was developed in Urdu for use by FOs in face-to-face consultations to facilitate informed decisions about cascade screening. Details of how the DeSIRe was developed can be found elsewhere [12]. Our research shows that the use of the DeSIRe would be acceptable to relatives of children with β-TM, but paradoxically, they would prefer the use of directive language and images [12]. To ensure alignment with international consensus for population genetic screening programmes [13], and ethical principles of genetic counselling [5, 14], the DeSIRe remained non-directive for use in clinical practice. Given preferences in Pakistan for the use of more directive language versus our decision to ensure the DeSIRe remains non-directive, this study aimed to explore relatives’ experiences and acceptability of using the DeSIRe in routine clinical practice for decision making about cascade screening.

## Method

### Training the FOs

Based on similar workshops [15], we developed a ‘skills-building’ 2-hour workshop and handbook to implement the DeSIRe in the PTPP. Nine FOs consenting to use the DeSIRe in routine practice attended this workshop, which was conducted in the PTPP head offices (Lahore), led by HJ in Urdu. During the workshop, the FOs were introduced to the concept of SDM, and the DeSIRe and how to use it within routine practice. Given the use of several regional spoken (first) languages and dialects, and low literacy rates, FOs were asked to read the DeSIRe during family meetings and to supplement the information in local languages. These trained FOs were asked to use the DeSIRe in their routine practice, and to subsequently recruit participants for the study.

### Participants

Purposive sampling was used to recruit relatives of children with β-TM. The inclusion criteria were: blood relatives aged 18 years or older, who had attended a family meeting in which the DeSIRe was used and had not previously had thalassaemia carrier testing.

Relatives were recruited by the trained FOs, in seven cities of the Punjab province, including Lahore, Sheikhupura, Nankana Sahab, Kasur, Gujranwala, Multan and Faisalabad. FOs initially contacted parents of children with β-TM at thalassaemia clinics asking them to arrange a family meeting at their home with their relatives to provide information about the prevention of β-TM (this is usual PTPP practice). FOs also explained the study and obtained these parents’ consent to recruit their relatives following the family meetings.

Each of the nine FOs recruited six relatives, one male and one female from three different families, following the family meetings during April to June 2021. FOs used a proforma to ensure potential participants met the inclusion criteria. Potential participants provided their details and were contacted by a researcher by telephone to gain consent and arrange an interview. Sixty-six relatives were approached, and 62 initially agreed to participate and provided their contact details. Two of these relatives were excluded because they had already had carrier testing. When arranging the interview, six relatives declined to participate mainly because they had changed their minds. Fifty-four relatives participated in the study. The sampling strategy ensured diversity in terms of gender, age and educational attainment.

### Procedure

Semi-structured telephone interviews were carried out by HJ, MF and WNA in Urdu or Punjabi. All three interviewers were multilingual in English, Urdu and Punjabi. The interviews explored relatives’ experiences of the use of the DeSIRe in the family meeting, including their satisfaction with the DeSIRe, understanding of its content, perceptions of its purpose and influence on decision making. The interviews lasted approximately 40 min, were audio-recorded, translated and transcribed in English by two researchers (MF and WNA). SA (also multilingual in English, Urdu and Punjabi) assured the quality of the interviews, translation and transcription by listening to audio-recorded interviews, reading the transcripts and conducting the analysis simultaneously.

### Analysis

Reflexive thematic analysis was used to guide data analysis [16, 17], underpinned by the ‘effectiveness’ element of the RE-AIM evaluation framework [18], to understand relatives’ experiences of the DeSIRe in routine clinical practice. This approach is compatible with applied health research [19], and can enable exploration of participants’ subjective experiences. The analysis involved six phases (see Table 1 for details), using both deductive and inductive approaches [16]. To initially classify and organise data, transcriptions were analysed using subheadings identified from the initial review of participants’ responses to the interview questions (deductive analysis using NVivo 12, Sage Publications). The interviews were semi-structured, so participants’ responses lead to similar themes. Following further review of the transcripts, themes were added, modified, merged and changed iteratively (inductive analysis). Researchers’ subjectivity is an important analytic resource in reflexive thematic analysis [20]. Therefore, SA (of Pakistani origin based in the UK) initially analysed the first 17 transcripts as the most experienced qualitative researcher with expertise on the research topic. The remaining interviews were analysed by WNA and MF, researchers based in Pakistan, to ensure inclusion of any cultural nuances from their perspective. During analysis, differences by gender, educational attainment and place of residence were explored but not found, as shown by the demographic information preceding quotes in the Results section. Six recurrent themes were identified.

**Table 1:** Process of coding and thematic analysis [16].

Phase 1: familiarisation with data	SA listened to the interview audio recordings in Urdu, read and re-read the transcripts in English. WNA and MF conducted the interviews, transcribed the data and read the transcripts
Phase 2: generating initial codes	SA initially generated codes (using NVivo 12) based on questions in the interview guide, and patterns of meaning beyond the scope of the interview guide identified by SA, WNA and MF during Phase 1
Phase 3: searching for themes	As a starting point, the initial codes were categorised according to the topics in the interview guide (deductive analysis)
Phase 4: reviewing potential themes	SA, WNA and MF reviewed, added, modified, merged and changed these initial themes as analysis progressed (inductive analysis), to better understand relatives’ experiences and perception of the DeSIRe for decision making
Phase 5: defining and naming themes	SA, WNA, MF and HJ discussed, refined and agreed the names and interpretations of the themes. This phase enabled inclusion of these researchers’ subjectivity as ‘a resource for knowledge production’ rather than a credibility and validity assessment [17], allowing a more nuanced understanding of the data in this international collaborative study
Phase 6^a^: producing the report	SA produced the first draft of the report, with the support of HJ and MA’s clinical expertise to draft the Discussion. All the authors contributed to reviewing and revising the manuscript

^a^Analysis involved moving back and forth between the phases.

## Results

Interviews were conducted with 54 relatives. Their demographic characteristics are presented in Table 2. None of the participants had previously had genetic counselling or thalassaemia carrier testing.

**Table 2:** Demographic characteristics of study participants (*n* = 54).

	Male *n* = 27	Female *n* = 27
*Age in years, mean (SD)*	32.85 (10.15)	30.62 (9.44)
*Participants’ education, N (%)*		
Up to and including matriculation level^a^	8 (29.63)	12 (44.44)
Above matriculation level	19 (70.37)	15 (55.55)
*Marital status, N (%)*		
Married	18 (66.7)	21 (77.8)
Unmarried	9 (33.3)	6 (22.2)
*Relationship with child with β-TM, N (%)*		
Cousin (first, second or third)	5 (18.5)	1 (3.7)
Uncle/aunt	19 (70.4)	23 (85.2)
Grandparent	2 (7.4)	1 (3.7)
Sibling	1 (3.7)	2 (7.4)

^a^Matriculation level is equivalent to UK GCSE level at around age 16 years.

### Qualitative findings

The qualitative findings are presented below with illustrative quotes from participants, attributed anonymously but with details of gender, educational attainment and place of residence. ‘Low education’ is defined as having up to ‘Matriculation level’ education (equivalent to the UK GCSE level), and ‘high education’ as having above Matriculation level education.

#### Reasons for attending and expectations of the family meeting

Participants were usually invited to the family meeting with the FO by parents of the child with thalassaemia major to consider ‘testing for thalassaemia’. Participants explained they believed the meeting was for FOs to raise awareness of β-TM, and were expecting to learn about the cause of the condition and how it could be prevented, but many were unclear about the relevance of testing for themselves before the meeting:

“We thought that the husband, wife and the child who is suffering should get tests. We didn’t know that other relatives should also get tests.” (R5, male, high education, Multan)

“(what was the purpose of that meeting?) I had no idea. I thought thalassemia is one of many diseases, not that it’s got anything to do with relatives.” (R35, male, low education, Lahore)

#### Use of the DeSIRe, and satisfaction with its content and delivery

Participants explained how the FOs provided a copy of the DeSIRe to all the attendees, then read through it and further explained its content. Participants were generally satisfied with the DeSIRe, and reported that the information was clearly presented, and easy to read and understand. Their comments show that the FOs’ expertise was important in enhancing their understandings of the DeSIRe. For example, FOs explained the DeSIRe using local dialects, using their own examples to explain the inheritance images, and by encouraging and answering questions:

“…the way of explaining makes the whole difference.” (R53, female, high education, Nankana Sahab)

“…he explained the information in an easier way, in Punjabi, like a teacher.” (R26, male, high education, Kasur)

“He explained… with examples, like if both parents are carriers and if the one parent is a carrier.” (R25, male, low education, Lahore)

“The FO even explained the pictures, which made it even easier to understand.” (R48, female, high education, Sheikhupura)

“The FO was able to asses our understanding through question and answers…” (R38, female, low education, Lahore)

Furthermore, participants believed that the DeSIRe was suitable for use with individuals with no education or low levels of literacy, because of the use of simple language and images:

“…there’s nothing complicated in it (the DeSIRe)… Even I can explain this disease to an illiterate person by using this leaflet.” (R7, male, high education, Kasur)

Participants also gave suggestions for using the DeSIRe beyond the family meeting, showing further evidence that they valued the intervention. They believed that relatives unable to fully understand the information could use it for further clarity at a later stage within their own social networks, and that it was also valuable for disseminating information to relatives unable to attend the meeting:

“…they can discuss it with others and show them the leaflet that this is what they are talking about.” (R23, female, high education, Gujranwala)

“I also gave copies of it (DeSIRe) to some family members to read… I hope that they will also learn about it and gain information.” (R12, female, low education, Sheikhupura)

Overall, participants believed that the DeSIRe enabled them to better understand β-TM, its inherited nature and the relevance of thalassaemia carrier testing for relatives.

#### Perceptions of the purpose of the DeSIRe and its main message

Participants generally understood that the main purpose of the leaflet was to enable them to make decisions about carrier testing, particularly before marriage. They reported the DeSIRe enabled them to understand that two carriers could have a child with β-TM. Hence, they believed that a key message was to avoid the marriage of two carriers, irrespective of marrying within or out of the family:

“…this condition runs in the family… relatives are more prone to it… they need carrier testing especially before getting married.” (R21, male, high education, Nankana Sahab)

“…there are also chances… when marrying outside the family. So carrier testing is important before getting married in either situation.” (R40, male, high education, Gujranwala)

While participants generally recognised pre-marital carrier testing as an important step in preventing β-TM, they also raised ethical, social and cultural issues for individuals testing positive prior to marriage. For example, they were anxious about disclosing an individual’s carrier status when arranging a marriage because of the adverse implications for the carrier’s marriage prospects:

“They (parents) should not arrange marriages for their children within their families until they do the tests.” (R24, Female, Low education, Kasur)

“2–3 people were upset that if they test positive then it will be a problem to get married in the family.” (R2, male, low education, Faisalabad)

Participants also raised some sociocultural concerns about such pre-marital carrier testing. They believed that asking potential spouses to have carrier testing could damage family relationships, particularly if it is not an acceptable option. Furthermore, communication about pre-marital testing would require disclosure of carrier status, which could lead to stigmatisation of carriers as ‘diseased’ and unsuitable for marriage:

“…my cousin, who is a carrier, got engaged and when we asked them to also get testing, they refused and said that ‘it’s a problem in your girl’…they ended the relationship saying that they didn’t want their children to get sick as well.” (R45, female, low education, Gujranwala)

If the marriage is called off, it’s a huge problem in our family…” (R10, female, low education, Multan)

#### Usefulness of the DeSIRe in decision making

Participants believed that the DeSIRe was an appropriate intervention, which supported them to make a decision about carrier testing. This may be because the DeSIRe enabled them to understand the inherited nature of the condition and the concept of thalassaemia carriers, hence the relevance of carrier testing for relatives. Participants largely believed that it was important to prevent thalassaemia major, and the DeSIRe had helped them decide to opt for carrier testing, and to encourage others to also consider it:

“I decided to get myself carrier tested after this meeting. And I will also ask my other relatives to consider it.” (R31, male, high education, Lahore)

Some participants also believed that the DeSIRe allowed consideration of personal values in decision making, including the pros and cons carrier testing. In this context, participants raised concerns about the stigma of a positive test result:

“…test should be done but there are also societal factors… if someone turns out to be positive and people get to know about it, maybe he will get depressed or stressed. Like he will think that others should not know about it. As if there is something very wrong in him.” (R5, male, high education, Multan)

#### Appropriateness of using the DeSIRe in a family meeting

Most participants also believed that a family meeting was appropriate for providing the information in the DeSIRe. This was mainly because it enabled a better understanding of information in relatives with little or no education, and those unable to articulate or hesitant to ask questions:

“…those who are uneducated, they listen but don’t ask questions, even if they don’t understand…in a group meeting, when one person asks questions, they also benefit.” (R1, male, low education, Faisalabad)

Participants believed that following a family meeting, relatives had the reassurance that someone else in the family could understand the information in the DeSIRe and could explain it to them. Comparatively, one-to-one meetings with relatives were considered less supportive due to lack of familiarity or rapport with the FOs.

Participants added that a family meeting using the DeSIRe enabled relatives to gain a shared understanding of how to prevent β-TM. The meeting created an opportunity for them to openly discuss potential approaches to prevention, and provided a supportive environment to make shared decisions about carrier testing, and the DeSIRe facilitated discussion of the sensitive issue of how prevention of the condition was related to carrier testing and marriage:

“FO explained it, but after he left, we also helped each other… mainly about the importance of testing before matchmaking. Even those who were quiet during the meeting discussed their ideas. So, we just discussed the leaflet.” (R9, male, high education, Multan)

“…they (relatives) can ask ‘should we do it or not?’… People cannot make decisions alone, they get scared.” (R2, male, low education, Faisalabad)

“Group meetings are better… family members get to understand the consequences of thalassemia together, so they can make a better decision in future regarding cousin marriages. Moreover, they can also discuss their differences in opinion…” (R28, female, high education, Kasur)

Nevertheless, some participants raised the issue of lack of autonomy. They were concerned that women and unmarried (younger) relatives may not voice concerns or raise questions in a family meeting, because this may be perceived as disrespectful to male relatives/elders:

“…an advantage of an individual meeting is that unmarried people who …do not speak up in a group, can ask about carrier testing for a particular person (potential spouse).” (R8, male, high education, Nankana Sahab)

“Some questions can be personal like if husband and wife are planning for a child, then I think individual meetings should be arranged…” (R18, male, high education, Gujranwala)

#### Wider availability and accessibility of the DeSIRe

Considering the DeSIRe easy to understand and accessible, many participants suggested making it more widely available via social media and the internet:

“…it is an era of technology, internet, WhatsApp, so educational videos about this must be posted on social media and internet.” (R1, male, low education, Faisalabad)

In addition, some participants expressed the need for female FOs. This was because they considered it inappropriate for women to sit in the same room as a male FO, and/or ask questions of a male FO, particularly about marriage or pregnancy:

“…because females can ask private question of female FOs more easily.”

(R46, male, high education, Sheikhupura)

“…they can comfortably ask personal questions…related to marriage…it might be more appropriate to have two groups…” (R42, female, high education, Lahore)

## Discussion

The findings show that the participants were satisfied with the content of the DeSIRe and its delivery by the FOs. Unlike our previous findings [12], the need for using directive language in the DeSIRe was not mentioned. This difference in findings may be because the participants’ in the previous study were asked about their perceptions of the content of the DeSIRe in focus group settings, whilst the present study was conducted following the use of the DeSIRe in routine practice. Use of the DeSIRe in practice by FOs will have enabled a complete experience of the intervention, because FOs provided further examples and explanations, used local languages where necessary and answered any questions. This may explain why participants believed they had sufficient support to make carrier testing decisions themselves, and did not express the need for directive advice. In addition, there were no differences in the findings between participants of different educational backgrounds or gender.

Furthermore, the structure and content of the DeSIRe was based on international (western) guidelines for developing a decision support intervention [7, 21], raising concerns about its appropriateness for a population in a LMIC. However, participants’ satisfaction with the structure and content of the DeSIRe supports the use of such guidelines for developing interventions for use in LIMCs.

Similar to other studies [22], participants believed that they had little awareness of the inherited nature of the condition prior to the family meetings, and did not necessarily understand the relevance of carrier testing for relatives. This may explain relatives’ general lack of motivation to seek carrier testing. Nevertheless, participants believed that the use of the DeSIRe improved their knowledge of the condition, its inheritance and the relevance of thalassaemia carrier testing for themselves. These findings suggest that the DeSIRe could improve relatives’ awareness, hence increase uptake of cascade screening. Nevertheless, further research is needed to prove the efficacy of the DeSIRe and its potential to reduce the public health burden of β-TM in Pakistan.

Moreover, individuals with low levels of education can experience difficulties in making health-related decisions [23]. However, participants in this study generally believed that the DeSIRe was accessible and suitable for use with relatives with little or no education. This may be attributable to three main factors. Firstly, low health literacy was considered throughout the development phase of the DeSIRe, ensuring use of plain language, images, logical presentation of information and visual appeal. Secondly, the DeSIRe was co-produced ‘with’ relevant stakeholders including service users (relatives) and providers (FOs), rather than ‘for’ them [24]. Thirdly, the FOs further improved communication by conversing in local dialects where necessary [25], and creating an empowering environment during the family meetings by encouraging questions [26]. Overall, the DeSIRe is appropriate for use by healthcare professionals in populations with low literacy rates.

Similar to other studies supporting a group approach to genetic counselling [27, 28], participants considered the DeSIRe appropriate for use within a family setting. This may be because the meeting enabled relatives to understand that thalassaemia was an issue for the wider family, not just the affected child and parents. Also, the meeting enabled relatives to gain a shared understanding of how to prevent β-TM, openly discuss potential approaches to prevention (including the sensitive issue of marriage) and provided a supportive environment to make shared decisions about carrier testing.

In addition, participants highlighted the importance of female FOs to deliver the DeSIRe, particularly in families where male/female segregation or seclusion of women is preferred. The PTPP currently only employs male FOs, mainly for practical reasons relating outreach work. However, our findings suggest the need to address this gender imbalance. Besides, gender diversity among the FOs could increase access to the PTPP services, enable provision of better ‘patient/relative’ choice and satisfaction, and further improve communication [29].

As in previous studies [30, 31], participants were willing to pass on genetic information for cascade screening, highlighting use of the DeSIRe beyond its intended use within family meetings by FOs. This finding shows that the DeSIRe could enable relatives to gain confidence in sharing genetic information. Further research is needed to explore the dissemination of the DeSIRe by relatives and parents of children with β-TM to understand the longitudinal impact of this intervention.

Overall, participants believed that the DeSIRe supported decision making about carrier testing, particularly before marriage, and understood that a key message of the intervention was to avoid the marriage of two carriers, whether marrying within or out of the family. Similar to other populations [32, 33], participants also raised social and cultural concerns for individuals testing positive, including stigma, limited marriage prospects and damage to family relationships. These findings suggest that perceptions of stigma and discrimination against thalassaemia carriers may be a barrier to seeking carrier testing. Nevertheless, participants’ perceptions of the importance of thalassaemia carrier testing to prevent β-TM suggests that the DeSIRe has the potential to dispel stigma and reduce discrimination of thalassaemia carriers through improved awareness. In addition, participants suggested making the DeSIRe widely available to the general population via social media, further reinforcing the need for improved awareness to reduce social stigma associated with being a thalassaemia carrier. Conversely, improved awareness at population level could also result in blame for genetic conditions [34]; therefore, further research is needed on the impact of educational interventions on stigma related to thalassaemia carrier testing.

Communication of genetic information is challenging, particularly in populations in which the practice of consanguineous marriage is customary, because of the potentially stigmatising impact of messages about recessive inheritance and cousin marriages [31, 35]. However, the findings show that the public health message to ‘avoid the marriage of two carriers’ was culturally sensitive and acceptable to participants in our study. This may be because the DeSIRe was developed in Pakistan where the practice of consanguineous marriage is a social norm. In addition, such interventions are rarely developed within LMICs, and nor is their usefulness explored in similar populations within western countries. So, further research could explore the cultural appropriateness of the DeSIRe for cascade screening in similar populations based in other (western) countries, such as, Pakistanis in the UK or Netherlands.

This study has limitations. None of the participants were illiterate, suggesting that selection bias may have occurred during recruitment by the FOs. While participants with low education were recruited, further research with illiterate participants is needed to fully understand the accessibility of the DeSIRe. Also, the study was conducted in only one of the five provinces in Pakistan, via a government-funded thalassaemia prevention programme. There is no such prevention programme in the other provinces. Instead, non-governmental organisations for thalassaemia take on the task of information dissemination for cascade screening. Further research is needed to explore the wider use of the DeSIRe, including the other four provinces of Pakistan, where the availability of such prevention programmes is limited, and in other LMICs. Moreover, the DeSIRe was delivered by male FOs only. Given that Pakistan is a patriarchal society, an understanding of the implication of delivering the intervention via female FOs is needed.

## Conclusions

Our findings show that the DeSIRe is appropriate for use by healthcare professionals in routine practice in a LMIC, and has the potential to facilitate SDM about cascade screening for thalassaemia. Further research is needed to prove the efficacy of the DeSIRe.

## Supplementary information

DeSIRe Interview Schedule

## Data Availability

The datasets generated and/or analysed during the current study are available from the corresponding author on reasonable request.
