# Supplementary material for: Cascade screening for beta-thalassaemia in Pakistan: relatives’ experiences of a decision support intervention in routine practice
Source: Eur J Hum Genet. 2021 Oct 4;30(4):406–12. doi: 10.1038/s41431-021-00974-y (PMC8991204; doi:10.1038/s41431-021-00974-y)
Supplement: Supplementary file 1 — DeSIRe Interview Schedule [file 41431_2021_974_MOESM1_ESM.docx]

**DeSIRe Study**

**Interview schedule for relatives**

**Contextual information:**

You attended a meeting with a PTPP FO

- Who asked you to attend the meeting and why?
- Why did you decide to attend the meeting?
- What did you think the meeting was going to be about?

**Description of how the PTPP FO provided the DeSIRe:**

The PTPP FO used a leaflet to provide information about thalassaemia carrier testing

- How did the FO use the leaflet to provide information?
  - Were copies of the leaflet given to people at the meeting?
  - How did the FO go through/read this leaflet? (section by section with breaks, or the whole leaflet?)
  - Do you think the FO was checking whether or not people at the meeting could understand the information in the leaflet? (If ‘yes’, how?)
  - Was the meeting too long, too short or about the right amount of time?
- Did you (or other relatives) have any questions/concerns and were they answered/addressed? (What questions or concerns were raised/expressed in the group meeting by relative?)
- What did you think about the information being provided to a group of relatives?

**Views about the content of the DeSIRe:**

- In your view, is there any information that was missing from the leaflet? (What? Why?)
- Were there any parts of the leaflet that you thought were unnecessary? (Which? Why?)
- What do you think about the language used in the leaflet? (Easy or difficult to read/understand)?
- What do you believe are other advantage or disadvantages of the meeting with the FO?

**Relatives’ understandings of the DeSIRe:**

- What do you think was the main message/aim of the leaflet?
- Was it easy or difficult for you to understand the information provided by the FO? (What was easy? What was difficult? Why? How could this be changed?)
- In your opinion, how easy or difficult is it for a person with little or no education to understand the information given in the leaflet?
- Based on the information provided by the FO:
  - What is Beta-thalassaemia major?
  - How would you describe a thalassaemia carrier?
- In your opinion, is thalassemia a condition that runs in families? (explore understandings of inheritance)
- What do you believe are the implications of a carrier marrying within the family?
- What do you believe are the implications of a carrier marrying outside of the family?

**Decision-making about carrier testing using the DeSIRe:**

- Has the information provided in the leaflet helped you to make a decision about carrier testing? (How?)
- Were any section particularly helpful in making a decision about carrier testing? Which and why?
- Which sections unnecessary for making a decision about carrier testing? Which and why?
- Do you think you have enough information to make a decision yourself about carrier testing? Why?
- What is your decision about thalassaemia carrier testing? (Do you intend to opt for or against carrier testing, or are you undecided? Why?)
- Do/did you feel pressure to make a certain decision? (What? Why?)
- Do you believe you have made the decision that is right for you?
- How easy or difficult was it for you to make the decision about carrier testing? (What was easy? What was difficult? Why?)
